# Response of Black-Capped Chickadees to House Finch *Mycoplasma gallisepticum*

**DOI:** 10.1371/journal.pone.0124820

**Published:** 2015-04-16

**Authors:** André A. Dhondt, Keila V. Dhondt, Wesley M. Hochachka

**Affiliations:** 1 Bird Population Studies, Laboratory of Ornithology, Cornell University, Ithaca, New York, 14850, United States of America; 2 Department of Microbiology and Immunology, College of Veterinary Medicine, Cornell University, Ithaca, New York, United States of America; Justus-Liebeig University Giessen, GERMANY

## Abstract

Tests for the presence of pathogen DNA or antibodies are routinely used to survey for current or past infections. In diseases that emerge following a host jump estimates of infection rate might be under- or overestimated. We here examine whether observed rates of infection are biased for a non-focal host species in a model system. The bacterium *Mycoplasma gallisepticum* is a widespread pathogen in house finches (*Haemorhous mexicanus*), a fringillid finch, but an unknown proportion of individuals of other songbird species are also infected. Our goal is to determine the extent to which detection of *M*. *gallisepticum* DNA or antibodies against the bacteria in a non-fringillid bird species is over- or underestimated using black-capped chickadees *Poecile atricapillus*, a species in which antibodies against *M*. *gallisepticum* are frequently detected in free-living individuals. After keeping black-capped chickadees in captivity for 12 weeks, during which period the birds remained negative for *M*. *gallisepticum*, four were inoculated with *M*. *gallisepticum* and four were sham inoculated in both eyes to serve as negative controls. Simultaneously we inoculated six house finches with the same isolate of *M*. *gallisepticum* as a positive control. All inoculated birds of both species developed infections detectable by qPCR in the conjunctiva. For the 6 weeks following inoculation we detected antibodies in all *M*. *gallisepticum*-inoculated house finches but in only three of the four *M*. *gallisepticum*-inoculated black-capped chickadees. All house finches developed severe eye lesions but none of the black-capped chickadees did. Modeling the Rapid Plate Agglutination test results of black-capped chickadees shows that the rate of false-positive tests would be not more than 3.2%, while the estimated rate of false negatives is 55%. We conclude that the proportion of wild-caught individuals in which we detect *M*. *gallisepticum*-specific antibodies using Rapid Plate Agglutination is, if anything, substantially underestimated.

## Introduction

Since the emergence of *Mycoplasma gallisepticum* in house finches *Haemorhous* (formerly *Carpodacus*) *mexicanus*, a species of finch in the family Fringillidae, during the winter of 1993–94 in Maryland, USA [1, 2] evidence has accumulated that diverse free-living bird species are infected by *M*. *gallisepticum*. While *M*. *gallisepticum* infections in five fringillid finch species was demonstrated by detection of the bacteria’s DNA [1, 3–5], documentation of infection of many other species is limited to positive tests for antibodies [6–8] or visual observations of birds with conjunctivitis at bird feeders [9]. Either of these two latter lines of evidence is weaker than detecting DNA, as false-positive results are possible [7, 10–12], but at unknown rates. Previous experimental infections with *M*. *gallisepticum* in the conjunctiva showed that all Fringillidae species tested developed physical signs, seroconverted, and that *M*. *gallisepticum* DNA could be recovered from the conjunctiva and/or from the choana for several weeks after exposure [6, 13–15]. In contrast to fringillids passerine birds belonging to other families rarely developed eye lesions, although they often seroconverted, and *M*. *gallisepticum* DNA could frequently be recovered from the conjunctiva and/or from the choana [6, 15]. The only species in which no evidence of successful infection was observed was the chipping sparrow [6]. The only non-fringillid experimentally infected species in which conjunctivitis was observed for extended periods (> 1 month) was the tufted titmouse (Paridae) [6]. In one of two experiments with house sparrow (Passeridae) only a transient mild conjunctivitis was observed in a single individual [15].

To provide a better understanding how non-fringillid bird species in North America respond to *M*. *gallisepticum* infection we inoculated a small number of black-capped chickadees *Poecile atricapillus* with *M*. *gallisepticum* isolated from a house finch and compared their response to that of house finches inoculated simultaneously with the same isolate. Our experiment differed from previous experimental infections in two ways: we conducted repeated pre-inoculation tests, and we used a control group of sham inoculated black-capped chickadees. The repeated testing of non-exposed birds permitted to determine the extent to which the Rapid Plate Agglutination test that we used to determine the presence of *M*. *gallisepticum*-specific antibodies would generate false positive results in black-capped chickadees. The repeated testing of birds known to have been inoculated allowed us to determine how long evidence of exposure of black-capped chickadees to *M*. *gallisepticum* could be detected, and compare this to the duration of infection in house finches, used as positive controls. We selected black-capped chickadees for our experiment based on their abundance at bird feeders that are suspected to be sources of transmission of the bacteria [16], the ease of maintaining them in captivity during the non-breeding season, and reports of conjunctivitis in black-capped chickadees [9]. Furthermore, in an earlier field study we found that in our study area 7% of 160 black-capped chickadees were seropositive for *M*. *gallisepticum* using the Rapid Plate Agglutination test, although we were unable to detect *M*. *gallisepticum* DNA from the conjunctival sack [8].

## Materials and Methods

### Ethics Statement

Wild birds were trapped using mist nets and cage traps under New York State Fish and Wildlife License 39 (Albany, NY) and permit 22669 from the United States Geological Survey, Department of the Interior (Laurel, MD). All care and sampling procedures were approved by Cornell University’s Institutional Animal Care and Use Committee (protocol 2006–094).

### Experimental birds and housing

In late fall 2013 we trapped 10 juvenile black-capped chickadees and six house finches in Tompkins County, New York (42°46’ N, 76° 45’ W) at bird-feeding stations baited with black-oil sunflower seeds. Trapped birds were color banded with individually unique combinations of color bands, kept in quarantine for 2 weeks, and then tested by qPCR and rapid plate agglutination tests for possible previous exposure to *M*. *gallisepticum*. We used a two-week quarantine period because our previous experience showed that infected birds will express infection under the stress of captivity within this timespan. Following quarantine, we placed five black-capped chickadees in each of two semi-outdoor aviaries (width 1.8m × length 3.6m × height 2.0m) with water, pelleted diet (Roudybush, Inc. Cameron Park, CA) mixed with sun-flower seeds (50:50) dispensed from a multiperch tube feeder and provided *ad libitum*. For each black-capped chickadee group an extra plastic dish with only sunflower seeds was placed on the floor. One artificial tree and multiple wooden perches were distributed inside each aviary, as well as ceramic heat lamps. Knowing that black-capped chickadees sleep individually in cavities during winter, six wooden nest boxes were placed inside each aviary. The six house finches were placed in an adjacent aviary (width 1.2m × length 2.1m× height 2.0m) with water, two multiperch tube feeders filled with a pelleted diet (Roudybush, Inc. Cameron Park, CA), perches and an artificial tree as well as ceramic heat lamps Eight black-capped chickadees remained available at the start of the experiment because two died from undetermined causes.

### *M*. *gallisepticum* tests

Sampling for detection of *M*. *gallisepticum* DNA was done by swabbing the conjunctiva of both eyes of a bird using a separate sterile cotton tipped 3 inch wood handle swab for each eye (Fisher Scientific) that was then placed in 200 μl tryptose phosphate broth (TPB) and stored at—25° C. DNA extraction from conjunctival swab samples was carried out using a Qiagen DNeasy blood and tissue kit (Qiagen, Valencia, California, USA), following the manufacturer’s recommended protocol for the purification of total DNA from animal tissues. Conjunctival swabs were tested for the presence of *M*. *gallisepticum* DNA using qPCR as described by Grodio et al. [17]. The test provides the number of mgc2 gene copies in the swab, hereafter called *Mycoplasma* load. The assay exhibited a detection limit that was below 14 copies per reaction based on tests using a plasmid standard and below 10 copies per reaction based on tests using a genomic DNA standard [17]. For antibody testing blood samples taken from a bird’s left brachial vein were collected into micro-capillary tubes. Serum was tested for *M*. *gallisepticum* antibodies by Rapid Plate Agglutination using commercially available *M*. *gallisepticum* antigen produced by Charles River Laboratories, Inc using the A5969 *M*. *gallisepticu*m poultry strain. We have used this procedure routinely since 2001 in our work on house finches [14–16, 18], which has permitted us to longitudinally follow the presence of antibodies in individual birds.

Prior to experimental inoculation all birds tested negative for *M*. *gallisepticum* antibodies for nearly 3 months in captivity; tests were conducted on December 17, 31 2013; January 15, 29; February 3, 13; and March 7 2014. On this last date a conjunctival swab sample was also taken from each bird and tested for the presence of *M*. *gallisepticum* DNA using qPCR to confirm the absence of M. gallisepticum DNA in the eye swabs of all birds. On day 3, 7, 14, 21, 28 and 42 post- inoculation (PI) birds were trapped, inspected for eye lesions that were scored on a scale of 0 (absence of lesions) to 3 (severe lesions)[19], and conjunctival swabs were taken for qPCR. Because in previous experiments antibodies were rarely detected earlier than two weeks post-inoculation we took the first blood samples on day 14 PI, and then on days 21, 28 and 42 PI. The serum was tested for *M*. *gallisepticum* antibodies by Rapid Plate Agglutination.

### Experimental procedure

Prior to inoculation the birds remained in captivity for 12 weeks. No black-capped chickadee or house finch used in our experiment showed evidence of previous exposure to *M*. *gallisepticum* by qPCR, Rapid Plate Agglutination, or physical signs of conjunctivitis. In one group every black-capped chickadee was inoculated on 10 March 2014 by instillation of droplets from a micropipette in each eye with 0.03 ml of *M*. *gallisepticum* inoculum NC2006 [20] (NCSU MDRL identifier: 2006.080-5-4p 1/09/09; 3.05×10^8^ CCU/ml), isolated by Dr. David Ley (Department of Population Health and Pathobiology, College of Veterinary Medicine, NC State University) from a sample collected from a symptomatic House Finch in Durham, NC. In our experimental infections we used a fourth culture passage. This strain was circulating in house finches in eastern USA around 2006, in which it was intermediate in virulence relative to other isolates that we have tested [21]. Our choice of this isolate for experimental infections of chickadees was motivated by our presumption that most infections of wild songbirds originated from *M*. *gallisepticum* infections of house finches; infection durations and associated rates of detection of infection would be relevant for interpretation of results from field surveys for the bacteria [21]. The inoculum was diluted 1:63 in Frey’s medium [22] to the target count of 4.82×10^6^ CCU/ml. Simultaneously we inoculated six house finches with the same inoculum. Because house finches (20–25 g) are almost twice the mass of black-capped chickadees (10-11g) we inoculated each house finch with 0.05 ml of the inoculum in each eye rather than with 0.03 ml. The black-capped chickadees in the negative control group were sham-inoculated with 0.03 ml of Frey’s medium in each eye. The experiment ended on 21 April, 42 days after inoculation, when all birds were humanely euthanized using CO_2_ (as approved by Cornell University’s Institutional Animal Care and Use Committee; protocol 2006–094) and necropsied to examine the internal organs of the birds for macroscopic lesions.

### Statistical analyses

While the patterns that we describe below are clear from summaries of the raw experimental data from our experiment (S1 Table), we also conducted statistical analyses to confirm that the observed patterns were unlikely to have been found by chance. Because of prior knowledge that both bacterial loads and probabilities of detecting antibodies will change through time post-inoculation[23], we conducted regression analyses in which we allowed the response to increase and potentially also decline again, using day post-inoculation in both linear and quadratic regression terms. All data from dates prior to inoculation were treated as being taken on day 0 post-inoculation (i.e. as though all samples had been taken immediately prior to experimental inoculation). For black-capped chickadees, for which both inoculated and uninoculated control birds were sampled, we wanted to look for statistically-detectable differences in responses in the period following inoculation of the experimental birds. We described these effects in statistical terms as inoculated × day_post-inoculation interactions (inoculated being a 0/1 variable), which would allow for responses to vary through time for inoculated birds while permitting different patterns of response (including no detectable response throughout the duration of the experiment) for the control black-capped chickadees. To allow for contrasting effects of inoculation between black-capped chickadees and house finches in some analyses, we used statistical species × inoculated × day_post-inoculation interactions.

qPCR analyses of samples from conjunctival swabs provide measures of the *Mycoplasma* load in each sample, and we log-transformed these qPCR values for use in generalized linear mixed models that assume a normal distribution of errors. Results from Rapid Plate Agglutination testing, in contrast, are binary, and therefore data from Rapid Plate Agglutination tests were analyzed as logistic regressions (generalized linear models assuming a binomial error).

Because each bird in the study was sampled multiple times, the measured responses are potentially non-independent. As a result, in all of our analyses we tried to fit a random effect of individual, allowing for either time-invariant differences in response among birds or time-varying difference using an auto-regressive random effect (AR(1) random effect covariance structure) using PROC GLIMMIX of SAS [24]. In no case did we find evidence of consistent difference among birds: for our normal-error models the estimated variance accounted for by the random effect was zero, and for models with binomial-error responses the models failed to converge to solutions when a random effect was present and so the results from logistic regressions that we present are from models without random effects.

## Results

### Response of black-capped chickadees to *M*. *gallisepticum* inoculation

**Fig 1:**
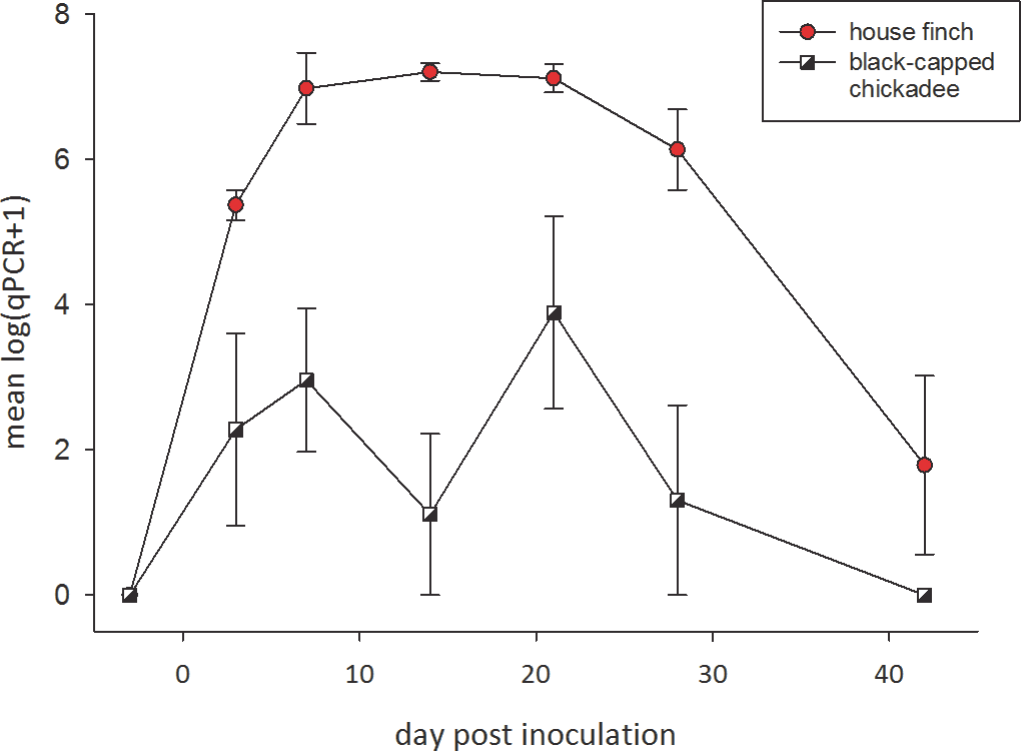
Mean number of copies of *M*. *gallisepticum* DNA detected in response to inoculation with a house finch strain of *M*. *gallisepticum* in conjunctival swabs of 6 house finches (circles) and 4 black-capped chickadees (squares). The 4 control black-capped chickadees sham-inoculated with Frey’s medium remained negative throughout (not shown). The values on the y-axis are the means log-transformed counts from qPCR with their standard errors.

All birds remained negative for antibodies on seven Rapid Plate Agglutination tests during the 81 days of the pre-inoculation period suggesting high test specificity. In none of the birds of either species did we detect *M*. *gallisepticum* DNA prior to inoculation. After sham-inoculation the negative control black-capped chickadees remained negative for *M*. *gallisepticum* by qPCR and Rapid Plate Agglutination test. After inoculation all four inoculated black-capped chickadees developed *M*. *gallisepticum* infections in the conjunctiva that were detectable by qPCR, but none developed physical signs. Upon necropsy none showed macroscopic lesions in any organ. Average black-capped chickadee *M*. *gallisepticum* load increased to day 7 and fluctuated to day 21 PI, after which it decreased to undetectable levels by day 42 PI (Fig 1). Even for birds in which bacterial DNA was detected, it was not consistently detected over the entire likely period of infection: we detected *M*. *gallisepticum* DNA on only 2 out of 6 sampling dates in three birds (days 3 and 7; 3 and 21; 7 and 21 PI), and in 4 consecutive dates in the fourth individual. (days 7, 14, 21, 28 PI) suggesting a low *M*. *gallisepticum* load in the conjunctiva of black-capped chickadees following experimental inoculation. *M*. *gallisepticum* DNA was no longer detected on day 42 PI. *M*. *gallisepticum*-specific antibodies were detected in only three of four black-capped chickadees, even though active infections by the pathogen were confirmed in all black-capped chickadees in our tests for presence of DNA, implying a low diagnostic sensitivity for the Rapid Plate Agglutination test in black-capped chickadees and/or a weak serological response.

Statistical tests confirm that the detection of antibodies to *M*. *gallisepticum* is unlikely to be the result of false-positives in Rapid Plate Agglutination tests. Changes in probability of a positive Rapid Plate Agglutination test are well described by a logistic regression model in which the probability of a positive test changes through time post-inoculation. The model used a statistical interaction to allow differences between control birds, for which no change in probability would be expected, and experimentally-inoculated birds. For inoculated birds, the change in probability of positive Rapid Plate Agglutination test was statistically-significant and non-linear, with the model containing both the linear regression term inoculated × day_post-inoculation (F = 14.39, df = 1, 84; P = 0.0003), and the quadratic term inoculated × day_post-inoculation^2^ (F = 14.39, df = 1, 84; P = 0.02). Further, the intercept of this model represents the background rate of false positive Rapid Plate Agglutination tests, which is only 3.2%. Conversely, this same model provides an estimated rate of false negatives—failures to detect antibodies from known-infected birds—to be minimally 32%. This chance is based on an estimated (least-squared mean) 55% chance (95% confidence interval: 32%- 77%) of detecting antibodies for an inoculated black-capped chickadee 42 days post-inoculation; day 42 was the sampling day with the highest estimated probability of detecting antibodies in blood from an inoculated black-capped chickadee.

### Comparison of the responses of black-capped chickadees and house finches to *M*. *gallisepticum* inoculation

House finches displayed a stronger and more rapid response to *M*. *gallisepticum* inoculation than black-capped chickadees by all measures of infection and disease (Fig 1). Following inoculation *Mycoplasma* load measured by qPCR increased more slowly in black-capped chickadees than in house finches, and the difference in rate of increase was statistically significant (in Table 1, the linear and quadratic inoculated × day_post-inoculation × species interactions). Among house finches average *Mycoplasma* load increased to 10^7^ detected copies at day 14 PI and remained at that level to day 21 PI, after which it decreased. All house finches reached 10^7^ detected copies of the mgc2 gene. Average *Mycoplasma* load in black-capped chickadees increased to day 7 PI, after which it fluctuated until day 21 PI, declining to become undetectable by day 42 PI. In the black-capped chickadees the highest value reached was either 10^5^ (n = 3) or 10^4^ (n = 1) detected copies, at least two orders of magnitude lower than in the finches. Additionally, all house finches developed severe conjunctivitis, having an eye score of 3 (the maximum [19]) in both eyes on day 21 PI, and three out of six maintained that eye score until the end of the experiment at day 42 PI. In contrast, none of the black-capped chickadees developed eye lesions.

**Table 1:** Results of a GLMM comparing log(*Mycoplasma* load) in conjunctival swabs of 6 captive house finches and 4 black-capped chickadees 3 days before inoculations, and on days 3, 7, 14, 21, 28 and 42 post inoculation.

Effect	Numerator degrees of freedom	Denominator degrees of freedom	F	P
Species	1	12	13.17	0.0035
Exposed x Day_PI	1	78	85.96	<.0001
Exposed x Day_PI x Day_PI	1	78	79.41	<.0001
Exposed x Day_PI x species	1	78	10.44	0.0018
Exposed x Day_PI x Day_PI species	1	78	9.08	0.0035

The *Mycoplasma* load increases more rapidly (significant two and three way interaction terms) and to a higher level (significant species effect, and Fig 1) in house finches than in black-capped chickadees. The second degree term was included to describe the non-linear response.

**Fig 2:**
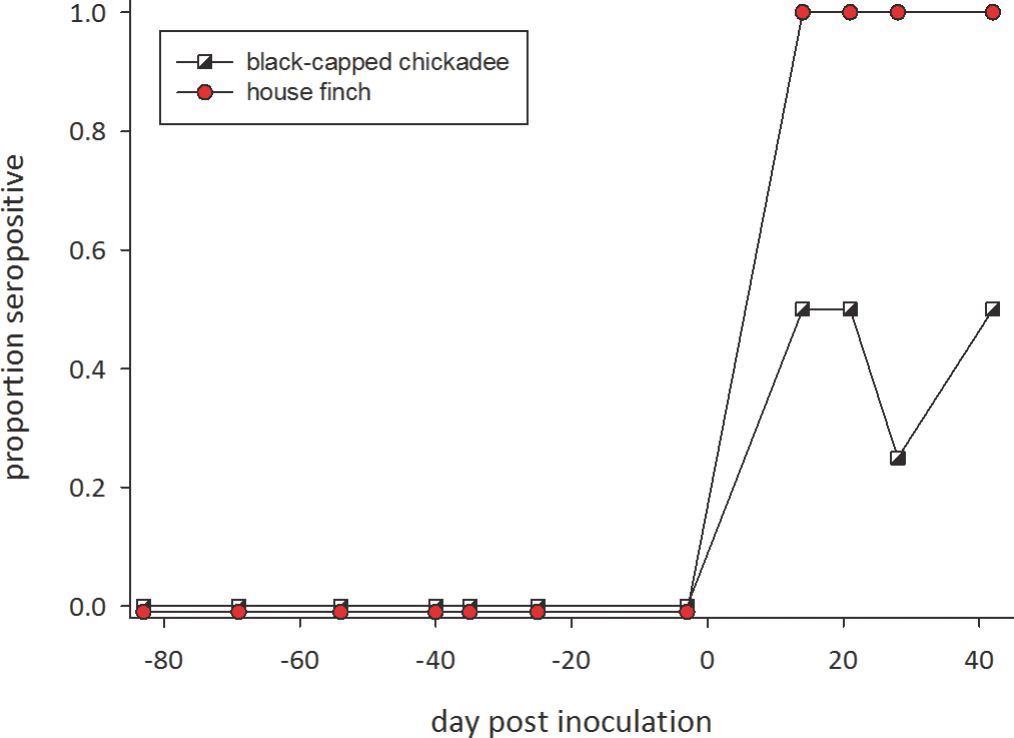
Proportion of individuals in which *Mycoplasma gallisepticum*-specific antibodies were detected using an RPA test after inoculation (on day 0) with a house finch strain of *M*. *gallisepticum* in 6 house finches (circles) and 4 black-capped chickadees (squares). The 4 control black-capped chickadees inoculated with Frey’s medium remained negative throughout (not shown). On each date birds were scored as having (1) or not having (0) antibodies.

As with levels of detected DNA, sensitivity of antibody tests was greater for house finches than for black-capped chickadees (Fig 2). Antibodies were detected in all house finches from 14 to 42 days post-inoculation. This difference in sensitivity was statistically significant (P<0.0001 for an inoculated × day_post-inoculation × species interaction in a logistic regression).

## Discussion and Conclusions

Our experiment shows that black-capped chickadees inoculated with house finch *M*. *gallisepticum* in the conjunctiva maintain an infection for several weeks and develop antibodies detectable by Rapid Plate Agglutination- in response to their infection. Although the experimental birds did not develop eye lesions Hartup [3] reported black-capped chickadees with conjunctivitis and we trapped one chickadee with mild unilateral conjunctivitis at our study site [8]. Among four tufted titmice, another species in the family Paridae, experimentally infected by Farmer et al. [6] two developed clinical disease while *M*. *gallisepticum* was detected by PCR from all four, and all seroconverted. These two prior studies suggest that Paridae exposed to *M*. *gallisepticum* sometimes develop eye lesions, but, in general, seem to be tolerant to *M*. *gallisepticum* infection,

Although black-capped chickadees were susceptible to infection by house finch-origin *M*. *gallisepticum*, the bacteria appear better adapted to grow in house finches than in chickadees. We see this in the shorter duration that *M*. *gallisepticum* was detectable in the conjunctiva of chickadees, the lower *Mycoplasma* load measured (Fig 1), and in the lower probability of detecting circulating antibodies (Fig 2).

As a consequence of the bacteria’s poorer ability to grow in black-capped chickadees, our experiment suggests that the proportion of wild black-capped chickadees that have been infected by *M*. *gallisepticum* will be under-estimated regardless of whether surveys use qPCR to detect DNA or test for the presence of antibodies by Rapid Plate Agglutination. More generally, our results suggest the potential that early stages of emergence of pathogens in novel hosts, or short-term but ultimately failed host transfers in new hosts, may be occurring at rates underestimated by surveillance for pathogens.

## Supporting Information

S1 Table Raw data of experiment. nd = no data; qPCR: number of copies of mg2 gene. (DOCX)
