# Supplementary material for: Response of Black-Capped Chickadees to House Finch Mycoplasma gallisepticum
Source: PLoS One. 2015 Apr 16;10(4):e0124820. doi: 10.1371/journal.pone.0124820 (PMC4400008; doi:10.1371/journal.pone.0124820)
Supplement: S1 Table — (DOCX) [file pone.0124820.s001.docx]

S1 Table – raw data of experiment.

nd = no data; qPCR: number of copies of mg2 gene.

| House finch - inoculated 10 March 2014 | | | | | | | |  | |  | |  | |  | |
| --- | --- | --- | --- | --- | --- | --- | --- | --- | --- | --- | --- | --- | --- | --- | --- |
| individual | date 2014 | | day PI | | eyescore L | | eyescore R | | RPA | | qPCR | | Log(mgc2+1) | |  |
| 1 | | 7-Mar | | -3 | | 0 | | 0 | | 0 | | 0 | | 0.00 | |
|  | | 13-Mar | | 3 | | 0 | | 0 | | nd | | 3.15E+05 | | 5.50 | |
|  | | 17-Mar | | 7 | | 2 | | 2 | | nd | | 3.23E+07 | | 7.51 | |
|  | | 25-Mar | | 14 | | 3 | | 3 | | 1 | | 3.30E+07 | | 7.52 | |
|  | | 31-Mar | | 21 | | 3 | | 3 | | 1 | | 9.62E+06 | | 6.98 | |
|  | | 7-Apr | | 28 | | 3 | | 3 | | 1 | | 3.49E+04 | | 4.54 | |
|  | | 21-Apr | | 42 | | 1 | | 1 | | 1 | | 0 | | 0.00 | |
|  | |  | |  | |  | |  | |  | |  | |  | |
| 2 | | 7-Mar | | -3 | | 0 | | 0 | | 0 | | 0 | | 0.00 | |
|  | | 13-Mar | | 3 | | 0 | | 1 | | nd | | 1.53E+05 | | 5.18 | |
|  | | 17-Mar | | 7 | | 2 | | 2 | | nd | | 4.12E+07 | | 7.62 | |
|  | | 25-Mar | | 14 | | 2 | | 3 | | 1 | | 2.31E+07 | | 7.36 | |
|  | | 31-Mar | | 21 | | 3 | | 3 | | 1 | | 7.54E+06 | | 6.88 | |
|  | | 7-Apr | | 28 | | 3 | | 3 | | 1 | | 8.99E+06 | | 6.95 | |
|  | | 21-Apr | | 42 | | 3 | | 3 | | 1 | | 0 | | 0.00 | |
|  | |  | |  | |  | |  | |  | |  | |  | |
| 3 | | 7-Mar | | -3 | | 0 | | 0 | | 0 | | 0 | | 0.00 | |
|  | | 13-Mar | | 3 | | 0 | | 0 | | nd | | 2.14E+06 | | 6.33 | |
|  | | 17-Mar | | 7 | | 2 | | 2 | | nd | | 2.59E+07 | | 7.41 | |
|  | | 25-Mar | | 14 | | 3 | | 3 | | 1 | | 1.95E+07 | | 7.29 | |
|  | | 31-Mar | | 21 | | 3 | | 3 | | 1 | | 4.68E+07 | | 7.67 | |
|  | | 7-Apr | | 28 | | 3 | | 3 | | 1 | | 3.73E+07 | | 7.57 | |
|  | | 21-Apr | | 42 | | 3 | | 3 | | 1 | | 0 | | 0.00 | |
|  | |  | |  | |  | |  | |  | |  | |  | |
| 4 | | 7-Mar | | 0 | | 0 | | 0 | | 0 | | 0 | | 0.00 | |
|  | | 13-Mar | | 3 | | 1 | | 0 | | nd | | 8.14E+04 | | 4.91 | |
|  | | 17-Mar | | 7 | | 3 | | 3 | | nd | | 1.56E+07 | | 7.19 | |
|  | | 25-Mar | | 14 | | 3 | | 3 | | 1 | | 2.12E+07 | | 7.33 | |
|  | | 31-Mar | | 21 | | 3 | | 3 | | 1 | | 5.67E+07 | | 7.75 | |
|  | | 7-Apr | | 28 | | 3 | | 3 | | 1 | | 6.97E+06 | | 6.84 | |
|  | | 21-Apr | | 42 | | 3 | | 3 | | 1 | | 1.72E+06 | | 6.23 | |
|  | |  | |  | |  | |  | |  | |  | |  | |
| 5 | | 7-Mar | | -3 | | 0 | | 0 | | 0 | | 0 | | 0.00 | |
|  | | 13-Mar | | 3 | | 0 | | 1 | | nd | | 1.93E+05 | | 5.29 | |
|  | | 17-Mar | | 7 | | 3 | | 2 | | nd | | 3.55E+04 | | 4.55 | |
|  | | 25-Mar | | 14 | | 3 | | 3 | | 1 | | 1.01E+07 | | 7.01 | |
|  | | 31-Mar | | 21 | | 3 | | 3 | | 1 | | 3.97E+06 | | 6.60 | |
|  | | 7-Apr | | 28 | | 3 | | 3 | | 1 | | 3.79E+06 | | 6.58 | |
|  | | 21-Apr | | 42 | | 3 | | 3 | | 1 | | 3.21E+04 | | 4.51 | |
|  | |  | |  | |  | |  | |  | |  | |  | |
| 6 | | 7-Mar | | -3 | | 0 | | 0 | | 0 | | 0 | | 0.00 | |
|  | | 13-Mar | | 3 | | 1 | | 1 | | nd | | 1.05E+05 | | 5.02 | |
|  | | 17-Mar | | 7 | | 2 | | 3 | | nd | | 3.74E+07 | | 7.57 | |
|  | | 25-Mar | | 14 | | 3 | | 3 | | 1 | | 5.15E+06 | | 6.71 | |
|  | | 31-Mar | | 21 | | 3 | | 3 | | 1 | | 6.31E+06 | | 6.80 | |
|  | | 7-Apr | | 28 | | 2 | | 2 | | 1 | | 2.04E+04 | | 4.31 | |
|  | | 21-Apr | | 42 | | 0 | | 0 | | 1 | | 0 | | 0.00 | |
|  | |  | |  | |  | |  | |  | |  | |  | |
| Black-capped chickadee : inoculated 10 March 2014 | | | | | | | |  | |  | |  | |  | |
| individual | |  | |  | |  | |  | |  | |  | |  | |
| 1 | | 7-Mar | | -3 | | 0 | | 0 | | 0 | | 0 | | 0.00 | |
|  | | 13-Mar | | 3 | | 0 | | 0 | | nd | | 0 | | 0.00 | |
|  | | 17-Mar | | 7 | | 0 | | 0 | | nd | | 6.70E+03 | | 3.83 | |
|  | | 25-Mar | | 14 | | 0 | | 0 | | 0 | | 0 | | 0.00 | |
|  | | 31-Mar | | 21 | | 0 | | 0 | | 0 | | 1.91E+05 | | 5.28 | |
|  | | 7-Apr | | 28 | | 0 | | 0 | | 0 | | 0 | | 0.00 | |
|  | | 21-Apr | | 42 | | 0 | | 0 | | 0 | | 0 | | 0.00 | |
|  | |  | |  | |  | |  | |  | |  | |  | |
| 2 | | 7-Mar | | -3 | | 0 | | 0 | | 0 | | 0 | | 0.00 | |
|  | | 13-Mar | | 3 | | 0 | | 0 | | nd | | 0 | | 0.00 | |
|  | | 17-Mar | | 7 | | 0 | | 0 | | nd | | 7.85E+03 | | 3.90 | |
|  | | 25-Mar | | 14 | | 0 | | 0 | | 0 | | 2.75E+04 | | 4.44 | |
|  | | 31-Mar | | 21 | | 0 | | 0 | | 1 | | 2.84E+04 | | 4.45 | |
|  | | 7-Apr | | 28 | | 0 | | 0 | | 0 | | 1.68E+05 | | 5.22 | |
|  | | 21-Apr | | 42 | | 0 | | 0 | | 1 | | 0 | | 0.00 | |
|  | |  | |  | |  | |  | |  | |  | |  | |
| 3 | | 7-Mar | | -3 | | 0 | | 0 | | 0 | | 0 | | 0.00 | |
|  | | 13-Mar | | 3 | | 0 | | 0 | | nd | | 2.29E+04 | | 4.36 | |
|  | | 17-Mar | | 7 | | 0 | | 0 | | nd | | 1.32E+04 | | 4.12 | |
|  | | 25-Mar | | 14 | | 0 | | 0 | | 1 | | 0 | | 0.00 | |
|  | | 31-Mar | | 21 | | 0 | | 0 | | 1 | | 0 | | 0.00 | |
|  | | 7-Apr | | 28 | | 0 | | 0 | | 1 | | 0 | | 0.00 | |
|  | | 21-Apr | | 42 | | 0 | | 0 | | nd | | nd | |  | |
|  | |  | |  | |  | |  | |  | |  | |  | |
| 4 | | 7-Mar | | -3 | | 0 | | 0 | |  | | 0 | | 0.00 | |
|  | | 13-Mar | | 3 | | 0 | | 0 | | nd | | 5.76E+04 | | 4.76 | |
|  | | 17-Mar | | 7 | | 0 | | 0 | | nd | | 0 | | 0.00 | |
|  | | 25-Mar | | 14 | | 0 | | 0 | | 1 | | 0 | | 0.00 | |
|  | | 31-Mar | | 21 | | 0 | | 0 | | 0 | | 6.78E+05 | | 5.83 | |
|  | | 7-Apr | | 28 | | 0 | | 0 | | 0 | | 0 | | 0.00 | |
|  | | 21-Apr | | 42 | | 0 | | 0 | | 1 | | 0 | | 0.00 | |
|  | |  | |  | |  | |  | |  | |  | |  | |
| Black-capped chickadee : sham inoculated 10 March 2014 | | | | | | | | | |  | |  | |  | |
| individual | |  | |  | |  | |  | |  | |  | |  | |
| 6 | | 7-Mar | | -3 | | 0 | | 0 | | 0 | | 0 | | 0.00 | |
|  | | 13-Mar | | 3 | | 0 | | 0 | | nd | | 0 | | 0.00 | |
|  | | 17-Mar | | 7 | | 0 | | 0 | | nd | | 0 | | 0.00 | |
|  | | 25-Mar | | 14 | | 0 | | 0 | | 0 | | 0 | | 0.00 | |
|  | | 31-Mar | | 21 | | 0 | | 0 | | 0 | | 0 | | 0.00 | |
|  | | 7-Apr | | 28 | | 0 | | 0 | | 0 | | 0 | | 0.00 | |
|  | | 21-Apr | | 42 | | 0 | | 0 | | 0 | | 0 | | 0.00 | |
|  | |  | |  | |  | |  | |  | |  | |  | |
| 7 | | 7-Mar | | -3 | | 0 | | 0 | | 0 | | 0 | | 0.00 | |
|  | | 13-Mar | | 3 | | 0 | | 0 | | nd | | 0 | | 0.00 | |
|  | | 17-Mar | | 7 | | 0 | | 0 | | nd | | 0 | | 0.00 | |
|  | | 25-Mar | | 14 | | 0 | | 0 | | 0 | | 0 | | 0.00 | |
|  | | 31-Mar | | 21 | | 0 | | 0 | | 0 | | 0 | | 0.00 | |
|  | | 7-Apr | | 28 | | 0 | | 0 | | 0 | | 0 | | 0.00 | |
|  | | 21-Apr | | 42 | | 0 | | 0 | | 0 | | 0 | | 0.00 | |
|  | |  | |  | |  | |  | |  | |  | |  | |
| 8 | | 7-Mar | | -3 | | 0 | | 0 | | 0 | | 0 | | 0.00 | |
|  | | 13-Mar | | 3 | | 0 | | 0 | | nd | | 0 | | 0.00 | |
|  | | 17-Mar | | 7 | | 0 | | 0 | | nd | | 0 | | 0.00 | |
|  | | 25-Mar | | 14 | | 0 | | 0 | | 0 | | 0 | | 0.00 | |
|  | | 31-Mar | | 21 | | 0 | | 0 | | 0 | | 0 | | 0.00 | |
|  | | 7-Apr | | 28 | | 0 | | 0 | | 0 | | 0 | | 0.00 | |
|  | | 21-Apr | | 42 | | 0 | | 0 | | 0 | | 0 | | 0.00 | |
|  | |  | |  | |  | |  | |  | |  | |  | |
| 9 | | 7-Mar | | -3 | | 0 | | 0 | | 0 | | 0 | | 0.00 | |
|  | | 13-Mar | | 3 | | 0 | | 0 | | nd | | 0 | | 0.00 | |
|  | | 17-Mar | | 7 | | 0 | | 0 | | nd | | 0 | | 0.00 | |
|  | | 25-Mar | | 14 | | 0 | | 0 | | 0 | | 0 | | 0.00 | |
|  | | 31-Mar | | 21 | | 0 | | 0 | | 0 | | 0 | | 0.00 | |
|  | | 7-Apr | | 28 | | 0 | | 0 | | 0 | | 0 | | 0.00 | |
|  | | 21-Apr | | 42 | | 0 | | 0 | | 0 | | 0 | | 0.00 | |
